# Probing Vasoreactivity and Hypoxic Phenotype in Different Tumor Grafts Grown on the Chorioallantoic Membrane of the Chicken Embryo In Ovo Using MRI

**DOI:** 10.3390/cancers14133114

**Published:** 2022-06-25

**Authors:** Johanna Buschmann, Dorothea M. Heuberger, Fatma Kivrak Pfiffner, Petra Wolint, Jae-Hwi Jang, Wolfgang Jungraithmayr, Pietro Giovanoli, Maurizio Calcagni, Conny F. Waschkies

**Affiliations:** 1 Center for Surgical Research, University Hospital Zurich, 8091 Zurich, Switzerland; petra.wolint@usz.ch (P.W.); conny.waschkies@usz.ch (C.F.W.); 2 Plastic Surgery and Hand Surgery, University Hospital Zurich, 8091 Zurich, Switzerland; pietro.giovanoli@usz.ch (P.G.); maurizio.calcagni@usz.ch (M.C.); 3 Institute of Intensive Care Medicine, University Hospital Zurich, 8091 Zurich, Switzerland; dorotheamonika.heuberger@usz.ch; 4 Institute of Medical Molecular Genetics, University of Zurich, 8091 Zurich, Switzerland; kivrakpfiffner@medmolgen.uzh.ch; 5 Department of Thoracic Surgery, University Hospital Zurich, 8091 Zurich, Switzerland; jaehwi.jang@hotmail.com (J.-H.J.); wolfgang.jungraithmayr@usz.ch (W.J.); 6 Department of Thoracic Surgery, Medical Center—University of Freiburg, Faculty of Medicine, University of Freiburg, 79106 Freiburg, Germany; 7 Institute for Biomedical Engineering, ETH and University of Zurich, 8093 Zurich, Switzerland

**Keywords:** chorioallantoic membrane (CAM), magnetic resonance imaging (MRI), A549 lung adenocarcinoma cell grafts, H460 lung adenocarcinoma cell grafts, MC-38 colon carcinoma cell grafts, hypercapnia, hyperoxia

## Abstract

**Simple Summary:**

Fertilized chicken eggs can be used to study tumors. During their development, chicken eggshells are fenestrated, and the chicken embryo that is enwrapped by a highly vascularized membrane becomes accessible. Tumor cells are then planted onto this membrane, which supports tumor growth and, after one week, the tumor graft is studied using magnetic resonance imaging. To characterize the tumor in living chicken embryos, a gas tube can be fixed into the eggshell window and the chicken embryo and hence, the tumor graft is exposed to air, carbon dioxide-enriched air, or oxygen enriched with carbon dioxide. Different tumor types react differently to such gas challenges, which can be quantitatively measured and related to the tumor grafts’ vascular functioning and oxygenation.

**Abstract:**

Tumor grafts grown on the chorioallantoic membrane (CAM) of chicken embryos represent a transition between cell culture and mammalian in vivo models. Magnetic resonance imaging (MRI) started to harness this potential. Functional gas challenge is feasible on the CAM. Using quantitative T1 and T2* mapping, we characterized the response of MC-38 colon, A549, and H460 adeno-carcinoma cell grafts to hypercapnic (HC) and hypercapnic-hyperoxic (HCHO) gas challenges, pertaining to the grafts’ vascular and oxygenation phenotypes. MR imaging revealed that larger T1 and T2* were located in the center of H460 and MC-38 tumors. Quantitative analysis showed a significant reduction in T1 and a significant increase in T2* in response to HCHO for A549 grafts, while H460 and MC-38 tumors did not respond to either gas challenge. Different tumor grafts respond differentially to HC and HCHO conditions. A549 tumor grafts, with higher vessel density and smaller tumor diameter compared with H460 and MC-38 grafts, had a significant response in T1 for HCHO and T2* increased slightly during HC and significantly under HCHO, consistent with a normoxic phenotype and functional vasoreactivity. Therefore, gas challenges enable differential characterization of tumor grafts with respect to their vascular and oxygenation status.

## 1. Introduction

Cancer is a worldwide burden, incurring high economical costs [1]. Historical and current prevention of cancer is based on scientific studies of the disease [2,3]. The preclinical studies of tumors are manifold, including the characterization of vascular and oxygenation phenotypes, and have currently produced a plethora of data, not only with respect to screening novel anticancer strategies [4,5], but also with regard to different types of preclinical animal models [6,7]. Although rodent models have been successfully used to answer diverse research questions [8,9], ethical considerations as well as the complex nature and organizational demands of experiments involving rodent models demand simpler models. In order to spare animals and costs, the chorioallantoic membrane (CAM) of the living chicken embryo provides a viable option to study angiogenesis in tumors [10], among others [11], particularly imaging modalities [12]. Moreover, it serves as an efficient and validated replace, reduce, refine (3R) model, and no veterinary license is needed until embryonic day 14 according to Swiss animal care guidelines. The CAM assay can be viewed as an intermediate between in vitro cancer cell cultures and in vivo (rodent) animal models [13] and can be easily imaged by magnetic resonance imaging (MRI) [14].

Tumor models grown on the CAM therefore represent a viable in vivo (in ovo) model [15]. MRI is only starting to be used to harness this potential, and tumor grafts and their metastases have been characterized morphologically [16,17].

Functional gas challenge is feasible on the CAM [10], when the chicken embryos are sedated with medetomidine [18]. In the present study, tumor phenotypes were characterized using quantitative T1 and T2* as readouts. We explored the response of MC-38 colon, and A549 and H460 adeno-carcinoma cell grafts to hypercapnic (HC) and hypercapnic-hyperoxic (HCHO) gas challenges, pertaining to the grafts’ vascular and oxygenation phenotypes [19]. We compared medetomidine [17] and a cooling sedation protocol for HCHO gas challenge. The hypotheses were that HC and HCHO gas challenges would evoke different responses in different tumor types. With MC-38 colon and H460 lung adeno-carcinoma, densely packed and less vascularized tumor types were chosen, in contrast to the more vascularized, granular and patchy A549 lung adeno-carcinoma. Moreover, we hypothesized that different gas challenges (HC and HCHO) would evoke different responses in T1 and T2* in a specific tumor type.

## 2. Materials and Methods

### 2.1. CAM Assay and Tumor Grafts

Experiments in chicken embryos until embryonic day 14 require no IACUC approval according to Swiss animal care guidelines (TSchV, Art. 112). Fertilized Lowman white LSL chicken eggs (Animalco AG Geflügelzucht, Staufen, Switzerland) were incubated at 37 °C and 65% relative humidity. On incubation day (ID) 3.5, 2 mL albumen was removed so that the developing CAM detached from the eggshell, and a circular window was excised into the eggshell.

Grafts from three cell lines were grown on the CAM from ID 7; A549 cells (ATCC, LGC Standards GmbH, Wesel, Germany), a human lung alveolar cancer cell line; as well as MC-38 (Kerafast, Boston, MA 02210, USA), a murine colon cancer cell line, syngeneic on a C57BL/6 background; and H460 cells (ATCC), a human lung cancer cell that has a growth rate almost double that of A549 cells. MC-38 and H460 cells were cultivated in DMEM (Life Technologies, Zug, Switzerland), supplemented with 10% FBS and 100 U/mL of penicillin and streptomycin, and incubated with 5% CO_2_ at 37 °C. A549 cells were cultivated in DMEM (Life Technologies), supplemented with NEAA, L-glutamine and 10% FBS, and incubated with 5% CO_2_ at 37 °C. The cells, harvested with trypsin (0.5%), were centrifuged and resuspended in serum-free DMEM. For tumor graft generation, MC-38 cells below passage 7 (P7), A549 cells below P13, and H460 cells below P8 were used. The cell suspension was 1:1 diluted with ice-cold growth factor-reduced Matrigel (Corning, Root, Switzerland GmbH, Switzerland) to a concentration of 0.5×10^6^ cells/50 μL. On a sterile petri dish, droplets of 50 μL of the cell-Matrigel suspension were formed and pre-warmed for 10 min at 37 °C. One such droplet was added on the CAM by a sterile 1 mL tip in the middle of a 1 cm diameter silicon ring to flatten the CAM surface and to aid as a landmark to locate the developing tumor grafts. Eggs were then further incubated until ID 14.

### 2.2. Magnetic Resonance Imaging

Before magnetic resonance imaging, eggs were cooled at 4 °C for 90 min for sedation [17]. Sedation by medetomidine in accordance with a previously published protocol [18] was compared for two kinds of tumor grafts (A549 and MC-38) and one gas challenge (HCHO, see Supplementary Materials Figure S1). MRI was performed on ID 14, 7 days after grafting in A549 (*n* = 14), H460 (*n* = 6), and MC-38 graft samples (*n* = 11) on a 4.7 T cm Bruker PharmaScan system (Bruker BioSpin, Ettlingen, Germany). An actively decoupled two-coil system, including a 72 mm quadrature resonator for excitation and a 20 mm single-loop surface coil for reception, was used for data acquisition. After cooling, the eggs were placed onto a custom-built sliding bed and wrapped in plastic tubing for isolation, retaining their temperature (approx. 4 °C) until the end of the experiment. All eggs survived.

MR images were acquired in 4 sagittal slices (FOV of 4.5 × 2.7 cm, spatial resolution of 300 × 300 µm^3^, slice thickness of 800 µm, interslice gap 200 µm) comprising the tumor graft with protocols including standard T1w FLASH sequences for anatomical reference, and T1 and T2* relaxometry protocols for quantitative T1 and T2* mapping. T1 and T2* relaxometry measurements were performed with a RARE sequence at variable repetition times (TR 430/800/1500/3000/4500 ms, RARE factor 2, TE 10 ms, and acquisition time 6 min) and a multi-echo gradient-echo (MGE) acquisition (TE 4-81 ms, echo spacing 7 ms, 3 averages, TR 1500 ms, and acquisition time 5 min).

Quantitative T1 and T2* are considered MRI markers associated with vascular functionality and oxygenation status when compared between periods of exposure to medical air, hypercapnia HC (5% CO_2_, 21% O_2_, balanced with N_2_), and hypercapnic-hyperoxia HCHO (5% CO_2_, 95% O_2_, i.e., carbogen). Gases were delivered through plastic tubing at 200 mL/min flow rate directed onto the CAM, which serves as a breathing organ during chicken embryo development. To prevent excessive loss of moisture, the eggshell window was kept covered by a sterile plastic plate, onto which the 20 mm single-loop surface coil used for MRI signal detection was attached for optimal signal sensitivity and through which the gas tube was guided.

### 2.3. Immunohistochemistry

For HIF-1-α staining of the excised, formalin-fixated, and paraffin-embedded H460 tissue, a mouse monoclonal antibody (abcam, ab16066, 1:1000) was used, after de-paraffinization, utilizing xylene before rehydration in a decreasing gradient of ethanol. Samples were briefly pre-treated in PT Link (DAKO) with Envision Flex Target Retrieval Solution high pH = 9.0 (DAKO, K8004), and then incubated with anti-HIF-1-α for 1 h. Then, secondary antibody was applied, consisting of labeled EnVision HRP/mouse (DAKO, K4001, dilution: RTU) for 20 min. After that, staining was performed in an Autostainer Link48 (DAKO), with Flex DAM and Substrate-Chromogen (DAKO, K3468) and ready-to-use Envision Flex Hematoxylin (DAKO, K8008).

For ki67 staining, H460 samples were pre-treated in PT Link (DAKO) with Envision Flex Target Retrieval Solution low pH (DAKO, K8005) and incubated with monoclonal mouse anti-human ki67 MIB-1 antibody (DAKO, IR626, dilution: RTU). Then, secondary antibody was applied, consisting of labeled polymer–HRP anti-mouse (DAKO, K4007, dilution: RTU). After that, staining was performed in an Autostainer Link48 (DAKO), with Flex DAM and Substrate-Chromogen (DAKO, K3468) and ready-to-use Envision Flex Hematoxylin (DAKO, K8008). Images of the stained sections were taken with a Leica 6000 light microscope (Leica, Basel, Switzerland) at 100× magnification. Histomorphometric analyses were based on three randomly located fields of view (FOVs) with a size of 100× 100 μm^2^. Cell densities of HIF-1α^+^ and ki67^+^ cells were calculated by division of cell number by area of FOV.

### 2.4. Data Analysis and Statistics

Quantitative T1 and T2* maps were computed from RARE images at multiple TR and MGE acquisitions at different TEs by exponential signal fitting (integrated in Brukers Paravision 5.1. MRI acquisition and reconstruction software). The graft response to the gas stimulus was assessed as the change in qT2* and qT1 values under HC and HCHO, respectively, compared with the baseline when the chicken embryo was exposed to medical air in a region of interest comprising the graft. Graphs and statistical analyses were produced by R v3.5.0 (R Foundation for Statistical Computing, Vienna, Austria), using a paired-samples Wilcoxon test for testing between conditions (air vs. HC and HCHO).

## 3. Results

**Figure 1:**
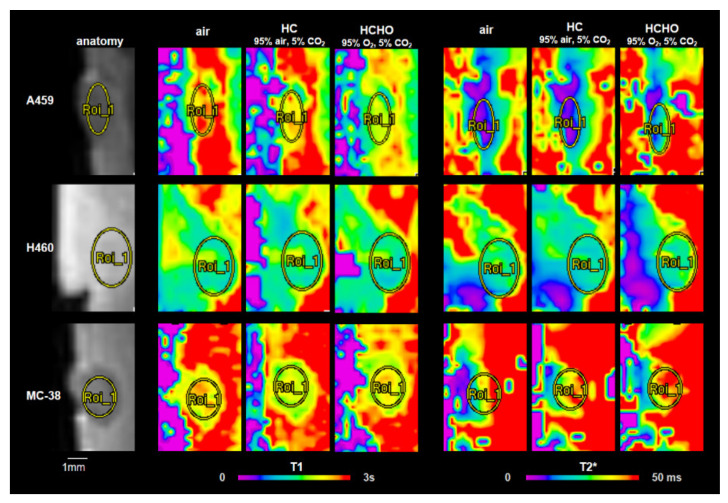
Representative MRI images of A549, H460, and MC-38 cell grafts grown on the CAM in ovo. Grafts are outlined on T1w anatomical reference images (left column) and quantitative color-coded T1 and T2* maps obtained while the graft was exposed to medical air, hypercapnia (HC), and hypercapnic-hyperoxia (HCHO). In H460 and MC-38 grafts, larger T1 and T2* were located in the graft center, revealing a distinct spatial distribution of T1 and T2* within the graft and response to gas challenge.

Quantitative T1 and T2* maps were obtained from all graft types displaying distinct spatial distribution patterns within the graft and functional response (Figure 1). Though of similar origin as the A549, H460 grafts more closely resembled the characteristics of MC-38 grafts in terms of graft size (~3.8 mm compared with ~4.0 mm in MC-38 grafts and ~2.0 mm in A549 grafts), spatial distribution of T1 and T2* (larger within the graft center for MC-38 and H460, and lower within the center for A549 grafts), and response to gas challenge.

**Figure 2:**
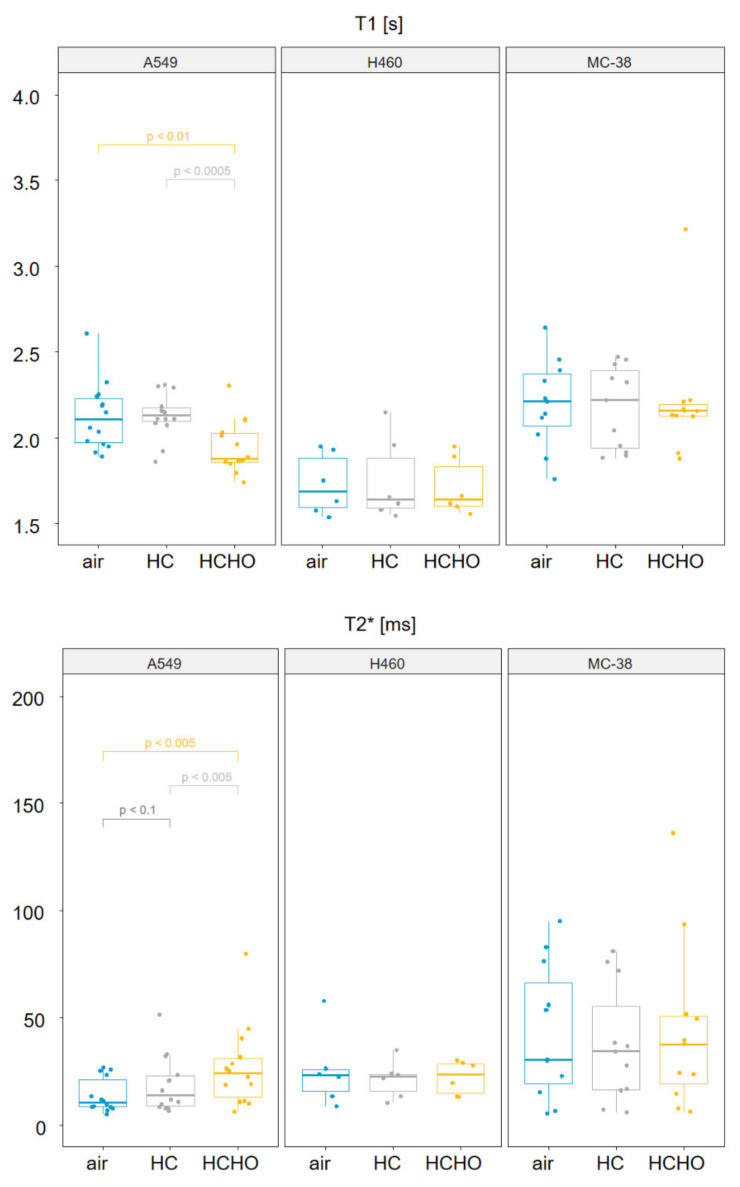
Quantitative T1 and T2* changes in response to hypercapnic (HC, grey) and hypercapnic-hyperoxic (HCHO, orange) gas challenge compared with medical air (blue), in A549, H460, and MC-38 grafts. In A549 grafts, T1 values significantly decreased in response to HCHO, but not to HC alone, as expected. T2* increased during HC, and even more so under HCHO. No significant change was revealed in either T1 or T2* in MC-38 or H460 grafts, respectively.

Region of interest analysis within the graft revealed a significant decrease in T1 (*p* < 0.01, Wilcoxon test) and increase in T2* (*p* < 0.005 Wilcoxon test) in the A549 grafts under hypercapnic-hyperoxia (HCHO) (Figure 2). Hypercapnia (HC) alone produced a weak effect (*p* < 0.1 Wilcoxon test) in T2* in the A549 grafts, but otherwise did not produce a significant response in the other tumor types. H460 or MC-38 grafts were not responsive to HCHO, consistent with their more hypoxic phenotype, which was previously corroborated with HIF-1α immunohistochemical staining for MC-grafts [10], and was accordingly found for H460 tumors in this study, with very similar and abundant quantities of proliferating ki67^+^ cells (Supplementary Materials Figure S2).

Moreover, a comparison to medetomidine sedation from a previous study [18] with the cooling sedation regimen revealed, for A549 grafts, that T1 reduction under HCHO challenge was only measured by trend (most individual data points displayed reduced response) because of high data variability under medetomidine, whereas cooling sedation resulted in a significant reduction in T1 for these grafts under HCHO (Supplementary Materials Figure S1). In contrast, for T2* assessment, both sedation regimen resulted in a significant increase under HCHO for A549 grafts (Figure 2 and Supporting Information S2).

## 4. Discussion

Quantitative T2* and T1 MR images were obtained from A549 and H460 lung adenocarcinoma as well as MC-38 colon carcinoma cell grafts (Figure 1). The results of region of interest analysis within the grafts revealed a significant decrease in T1 for the A549 grafts under hypercapnic-hyperoxia (HCHO), but did not change significantly in H460 or MC-38 grafts. On the other hand, T2* increased slightly during HC and significantly under HCHO in A549 grafts, but remained unaffected in the H460 and MC-38 grafts (Figure 2).

The CAM serves as a breathing organ during chicken embryo development. It is important to choose sedation protocols that interact as little as possible with gas challenge conditions. Under medetomidine anesthesia [18], A549 grafts revealed increased T2* values upon hypercapnic-hyperoxia (Supplementary Materials Figure S1), while MC-38 grafts displayed no such trend, such as under the present cooling protocol for sedation (Figure 2). However, T1 was significantly reduced under cooling in A549 grafts (Figure 2), an effect not observed under medetomidine (Supplementary Materials Figure S1), although graft size and T1/T2* heterogeneity were similar to those reported in the previous study [10]. Hence, differential effects arising from sedation protocols have to be considered, particularly if medetomidine is used, which transiently leads to higher arterial blood pressure and may therefore impact MRI readouts as well. Most likely, however, we suggest that the increased variance in the T1 values due to residual motion under medetomidine sedation may have contributed to a less consistent result in T1 in our previous study [10], and is in accordance with the longer scan times involved in qT1 assessment (compared with qT2* measurement).

O’Connor et al. reported that T1 is dependent on the concentration of molecular oxygen dissolved in plasma [20]. Accordingly, normoxic tissue that receives a 100% oxygen gas challenge would respond with a decrease in T1 caused by increases in dissolved plasma oxygen, while oxy-hemoglobin (HbO_2_) concentration would remain unaltered as it is fully oxygen-saturated before hyperoxic challenge. In contrast, if tumor tissue is locally hypoxic, as implied in the HIF-1α immunohistochemical staining for MC-38 [10] and H460 grafts, with many regions of HIF-1α-positive cells (Supplementary Materials Figure S2), and where hemoglobin is not fully oxygen-saturated before hyperoxic challenge, T1 is expected to be unaffected. These considerations are supported by our findings. On the other hand, the small, granular, and highly vascularized A549 grafts responded with a decrease in T1 under HCHO conditions, while the more homogenous, densely packed H460 and MC-grafts, both relatively large and less vascularized than A549 grafts, remained unaffected in their T1 response toward HCHO gas challenge.

As for the impact of gas challenge on T2*, Ganesh et al. reported in a rat MRI study of abdominal organs that hypercapnia with 5% CO_2_, in accordance with a reduced ratio of HbO_2_/Hb, led to a decreased T2* signal, which was clearly pronounced in the liver (−37%) and kidney cortex (−48%) and, to a lesser extent, in the paraspinal muscle (−5%) [21]. In our tumor grafts grown on the CAM, we did not observe such reduction in T2* under HC, but rather an increase in T2* in the A549 grafts, consistent with functional vasoreactivity under normoxic tumor conditions. This was validated in the healthy brain where breath-holding-induced hypercapnia was used to test the vasoreactivity [22].

In contrast, HCHO significantly increased T2* in the small, granular A549 grafts, while the more densely packed H460 and MC-38 grafts remained unaffected in their T2* response. According to Ganesh et al., hyperoxic conditions do not change the arterial oxygen saturation status, which is fully saturated for normoxic tissue, but increases venous oxygenation status with increasing HbO_2_/Hb ratio [21]. As a consequence, T2* increases under HCHO conditions, which was found for the A549 grafts that could be described as having a normoxic tumor phenotype. For the rather hypoxic H460 (Supplementary Materials Figure S2) and MC-38 grafts [10], the effect of vasoconstriction through hyperoxia was counteracting the expected increase in T2* observed for A549 tumors, or supporting vascular vessels displayed different vasoreactive potential. This may have led to the lack of significant changes in T2* for H460 and MC-38 grafts.

## 5. Conclusions

Our study consolidated previous findings in another lung adeno-carcinoma graft type (H460) and under a cooling sedation scheme. Different tumor grafts were characterized by a differential response to hypercapnic and hypercapnic-hyperoxic functional gas challenge. We found that in A549 tumor grafts, a significant response in T1 occurred during HCHO, consistent with the normoxic phenotype of the graft in comparison with the other rather hypoxic graft types. Furthermore, the response of A549 grafts under HC and HCHO in T2* pertained to its vascular reactivity. We therefore demonstrated the capability to use functional gas challenge approaches to noninvasively study vascular functional and oxygenation phenotypes of grafts in the CAM model.

## Data Availability

Not applicable.

## Supplementary Materials

The following supporting information can be downloaded at: https://www.mdpi.com/article/10.3390/cancers14133114/s1, Figure S1: Comparison of sedation protocols and their impact on HCHO gas challenge for A549 and MC-38 tumor grafts; Figure S2: Immunohistochemical staining of H460, A549, and MC-38 tumor tissues grown on the CAM.
