# Supplementary material for: Probing Vasoreactivity and Hypoxic Phenotype in Different Tumor Grafts Grown on the Chorioallantoic Membrane of the Chicken Embryo In Ovo Using MRI
Source: Cancers (Basel). 2022 Jun 25;14(13):3114. doi: 10.3390/cancers14133114 (PMC9265041; doi:10.3390/cancers14133114)
Supplement: Supplementary file 1 [file cancers-14-03114-s001.zip › cancers-1783862-supplementary.pdf]

cancers
MDPI

Supplementary Material

Probing Vasoreactivity and Hypoxic Phenotype in Different Tumor Grafts Grown on the Chorioallantoic Membrane of the Chicken Embryo in ovo Using MRI

Johanna Buschmann [1,2,*], Dorothea M. Heuberger [3], Fatma Kivrak Pfiffner [4], Petra Wolint [1,2], Jae-Hwi Jang 5, Wolfgang Jungraithmayr [5,6], Pietro Giovanoli [2], Maurizio Calcagni [2] and Conny F. Waschkies [1,7]

1 Center for Surgical Research, University Hospital Zurich, 8091 Zurich, Switzerland; petra.wolint@usz.ch (P.W.); conny.waschkies@usz.ch (C.F.W.)
2 Plastic Surgery and Hand Surgery, University Hospital Zurich, 8091 Zurich, Switzerland; pietro.giovanoli@usz.ch (P.G.); maurizio.calcagni@usz.ch (M.C.)
3 Institute of Intensive Care Medicine, University Hospital Zurich, 8091 Zurich, Switzerland; dorotheamonika.heuberger@usz.ch
4 Institute of Medical Molecular Genetics, University of Zurich, 8091 Zurich, Switzerland; kivrakpfiffner@medmolgen.uzh.ch
5 Thoracic Surgery, University Hospital Zurich, 8091 Zurich, Switzerland; jaehwi.jang@hotmail.com (J.-H.J.); wolfgang.jungraithmayr@usz.ch (W.J.)
6 Department of Thoracic Surgery, University Hospital Freiburg, 79117 Freiburg, Germany
7 Institute for Biomedical Engineering, ETH and University of Zurich, 8093 Zurich, Switzerland
* Correspondence: johanna.buschmann@usz.ch; Tel.: +41-44-2559895

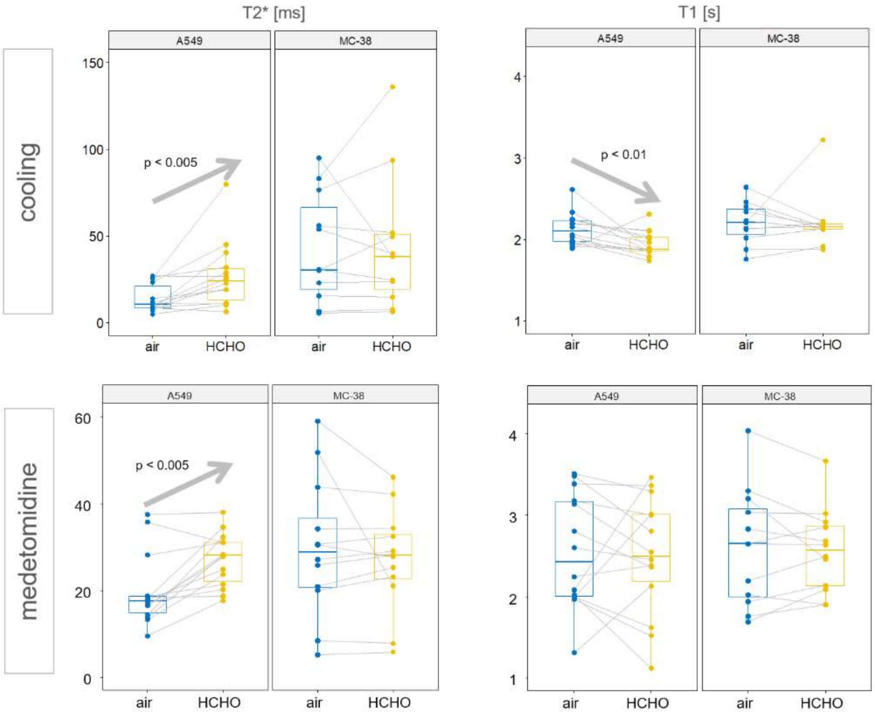

Figure S1. Comparison of sedation protocols and their impact on HCHO gas challenge for A549 and MC-38 tumor grafts. Sedation with medetomidine was compared with sedation by cooling at 4 °C. Impact on T1 and T2* for selected samples was assessed. *p* values refer to Wilcoxon test statistics; arrows indicate observed trends and changes upon gas challenge.

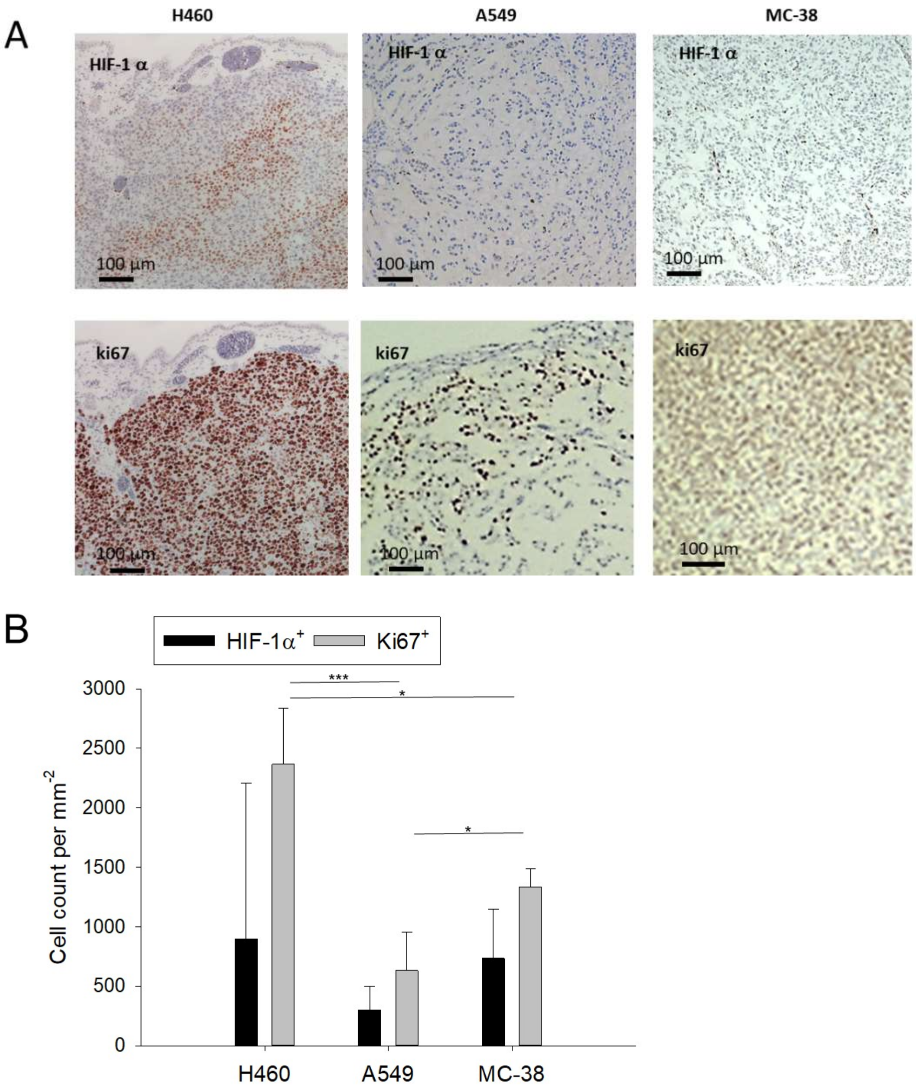

Figure S2. Immunohistochemical staining of H460, A549 and MC-38 tumor tissues grown on the CAM. Image taken at 100× magnification for a representative field of view. In the core tissue of H460 and MC-38 tumors, hypoxic areas are visible (numerous HIF-1*α*-positive brown cells), while in A549 tumors, only a few HIF-1α^+ cells can be seen. ki67, as a proliferation marker, shows that practically all H460 and MC-38 cells were proliferating (ki67-positive brown cells), while the pattern for A549 tumors was patchier in this regard (**A**). Quantitative assessment revealed no significant differences in HIF-1α^+ cells per area, while ki67$^+$ cell density was significantly different for all three tumor types (**B**). One-way ANOVA; $p < 0.05$ (*), $p < 0.01$ (**) and $p < 0.001$ (***).
